# fNIRS dataset of motor hand-gripping activity using the NIRSport2 system

**DOI:** 10.1117/1.NPh.13.S3.S32604

**Published:** 2026-09-16

**Authors:** Jamila Akhter, Hammad Nazeer, Noman Naseer

**Affiliations:** Air University Islamabad, Neuroimaging Research Group, Mechatronics and Biomedical Engineering Department, Islamabad, Pakistan

**Keywords:** functional near-infrared spectroscopy (fNIRS) dataset, brain-computer interface (BCI), motor activity, hand gripping

## Abstract

**Significance:**

The functional near-infrared spectroscopy (fNIRS) dataset acquired with the NIRSport2 device provides noninvasive recordings. The analysis of the fNIRS dataset can be used to refine existing models or propose new models to understand the motor cortex during voluntary motor activities, such as neuroplasticity and task-specific neural activation patterns.

**Aim:**

The hand-gripping dataset provides open-access fNIRS recordings of motor task-related brain activity to support the development and refinement of signal processing and machine learning models in brain–computer interface (BCI) research.

**Approach:**

Twenty healthy right-handed participants’ fNIRS data is acquired during a hand-gripping task from the motor cortex using an 8×8 optodes configuration (twenty channels) following the international 10/20 system. The NIRSport2 device (NIRx Medizintechnik GmbH, Germany) is used to record hemodynamic cortical activity with a sampling frequency of 10.1725 Hz in the form of light intensity. Signal processing software nirsLAB (version: v201904_64bit) is used for preprocessing and converting the light intensity into optical density, which is then converted into hemoglobin concentration changes (oxy- and deoxyhemoglobin) and finally filtered out for physiological artifacts, consistent with our previous work on this dataset.

**Results:**

This dataset is intended to explore patterns of brain activation during hand gripping, contributing to research on rehabilitation, motor learning, and neuroplasticity, and could be used to develop and validate classification algorithms, contributing to the field of BCI.

**Conclusions:**

These results demonstrate that the dataset is reliable and suitable for motor task analysis, benchmarking, and fNIRS-based machine learning studies.

## 1 Introduction

Brain–computer interface (BCI) systems are direct communication between machines and the human brain and are used to restore motor functions in impaired populations.^1^^,^^2^ The same principle extends beyond rehabilitation: in healthy users, BCIs can enable intuitive interaction with robots and other devices by bridging cortical signals and the environment.^3^ Consequently, BCIs are increasingly relevant to assistive technologies for conditions such as stroke, spinal cord injury, and neurodegenerative disease.^4^^,^^5^ Because voluntary movement intentions are encoded in motor cortex activity, signals from this region are particularly valuable for improving the reliability and practical impact of BCI systems.^6^

Neuroimaging methods capture brain activation as electrical, magnetic, or optical signals; common examples include electroencephalography (EEG), functional magnetic resonance imaging (fMRI), and fNIRS.^7^^–^^9^ These neuroimaging modalities provide information about different brain regions’ interaction and activation. EEG provides information about brain activation in the form of electrical activity,^10^ fMRI measures brain activity in the form of changes in blood flow^11^ and fNIRS provides information in the form of hemoglobin (both oxygenated and deoxygenated) concentration changes in the cerebral cortex in response to neural activation.^12^ fNIRS is an emerging neuroimaging technology used to acquire neural signals noninvasively to control BCI systems. Where fNIRS provides high spatial resolution to capture neural activation patterns in response to imaginary and motor activities.^13^

In fNIRS-based BCI, motor imagery (MI) and motor execution (MA) datasets have been successfully used to control several devices, including neurostimulators for cognitive enhancement,^14^ wheelchair control,^15^ smart prosthetic limbs,^14^ etc. The benefit of these fNIRS-BCIs applications is the noninvasive nature of the fNIRS signal acquisition with good spatial resolution.^16^ A drawback of the fNIRS signal acquisition is the low temporal resolution and signal-to-noise ratio.^17^ To improve the quality of the fNIRS-BCI application, signal processing methods are developed to extract meaningful information from recorded signals.^18^ The major step in the fNIRS signal processing for BCI application includes noise filtration, including physiological, motion, and instrumental noise, alongside signal correction, such as detrending and baseline correction, removal of noisy channels, selection of the channels with useful information about brain activation,^19^ and optimal feature extraction^20^ from the raw dataset. The data preprocessing requires algorithms to decode user intentions and improve the quality of the signal. Over the past several years, a number of techniques and algorithms have been employed for signal preprocessing and classification purposes.^21^ The application of algorithms provides subject-wise and activity-wise optimization to develop state-of-the-art fNIRS-BCI systems.^22^

Processing the neuroimaging dataset to decode brain activity requires a large amount of the training dataset for machine learning.^23^ In recent years, several studies have contributed fNIRS-BCI datasets to support algorithm training.^24^ However, fNIRS-BCI applications remain limited because of the small number of fNIRS neuroimaging devices and the scarcity of publicly available datasets.

Motivated by this data shortage, we acquired our own dataset by recording signals during motor execution (hand gripping). This work presents and provides an open-access dataset intended for the research community to support a wide range of applications in BCI research.

## 2 Experimental Design, Materials, and Methods

### 2.1 Participants

For this experimental data collection, a total of twenty right-handed participants are enrolled, consisting of 10 male and 10 female participants. To ensure a homogenous age group for this dataset, selected participant ages are in the range of 20±1.7  years. To ensure consistency and reliability, participant selection is done after confirmation of the absence of any neurological conditions.

Furthermore, participants are instructed to avoid caffeine consumption for at least 4 h before data collection. Experimental data collection is approved by the institutional review board at Air University, Islamabad, and written consent for the experimental procedure and use of data for research is taken from each subject. This dataset is documented under the ethical approval number: AU/EA/2022/02/011. Besides that, this experimental dataset collection follows ethical guidelines in the international set of ethical principles, which is the most recent version of the Declaration of Helsinki. By these considerations of methodological and ethical standards, the collected data provide consistent and useful insight into the brain’s hemodynamic response to motor activity and are both scientifically valid and ethically sound. Demographic details of all participants are given in Table 1.

**Table 1:** Summary of demographic information for all participants in the hand-gripping fNIRS dataset. All participants are right-handed with no reported neurological or psychiatric disorders.

S. No.	Gender	Age	Education
1.	F	20	Undergraduate
2.	F	21	Undergraduate
3.	M	19	Undergraduate
4.	F	18	Undergraduate
5.	F	20	Undergraduate
6.	M	23	Graduate
7.	M	20	Undergraduate
8.	F	19	Undergraduate
9.	M	20	Undergraduate
10.	M	21	Undergraduate
11.	M	22	Graduate
12.	F	21	Undergraduate
13.	F	23	Graduate
14.	M	22	Graduate
15.	M	21	Undergraduate
16.	F	20	Undergraduate
17.	M	19	Undergraduate
18.	F	20	Undergraduate
19.	M	22	Diploma of Associate Engineering (DAE )
20.	F	21	Undergraduate

### 2.2 Experimental Paradigm/Protocol

**Fig. 1:**
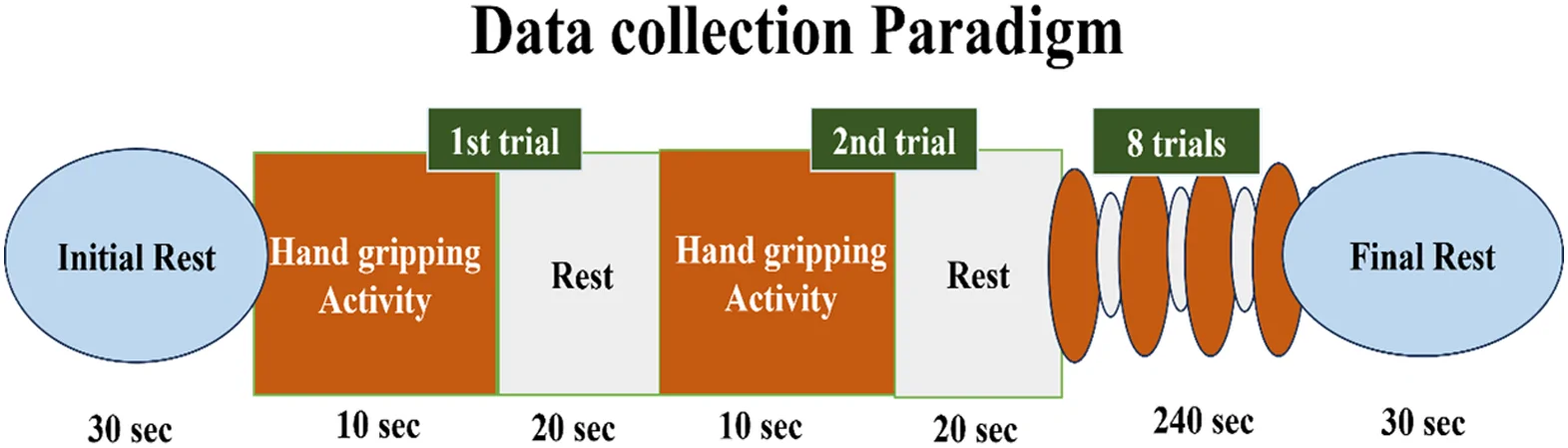
Experimental paradigm for hand-gripping activity data collection. The paradigm consists of 30-sec initial rest, 30-sec final rest, and 10 trials of activity versus rest (10-sec activity and 20-sec rest).

The experimental paradigm designed to capture neural activity during the performance of a motor task versus rest experiment is illustrated in Fig. 1. The paradigm consists of ten trials of alternate phases of activity (right-hand gripping) and rest to record brain function in response to motor activity. The experimental paradigm begins with an initial 30-sec resting period, during which the subject remains relaxed and still, creating a baseline period and establishing a reference for the motor task. Following the 30 sec initial rest, ten trials of activity and rest periods are started. Each trial consists of the 10 sec activity phase where participants engage in the motor task, involving hand-gripping, and the objective is to capture neural responses associated with voluntary activity. In each trail, 10 sec activity is followed by 20 sec rest phases, where participants return to the rest state to allow the brain to return to baseline. At the end of these ten trials, 30 sec final rest is implemented to again capture the brain activity baseline. In this way, the total duration for each participant is 360 sec (6 min).

### 2.3 Experimental Setup

For experimental data collection, we used the continuous-wave imaging system, called the NIRSport2 system, developed by NIRx Medical Technologies. In this study, the fNIRS system was used to measure cortical hemodynamic responses during motor execution tasks, which serve as an indirect marker of neural activation. NIRSport2 is provided with a montage consisting of eight emitters and eight detectors, placed onto the scalp surface and following a 10 to 20 standard system. The International system of electrode placement 10 to 20 ensures accuracy and consistency in optode positioning onto the skull surface.

**Fig. 2:**
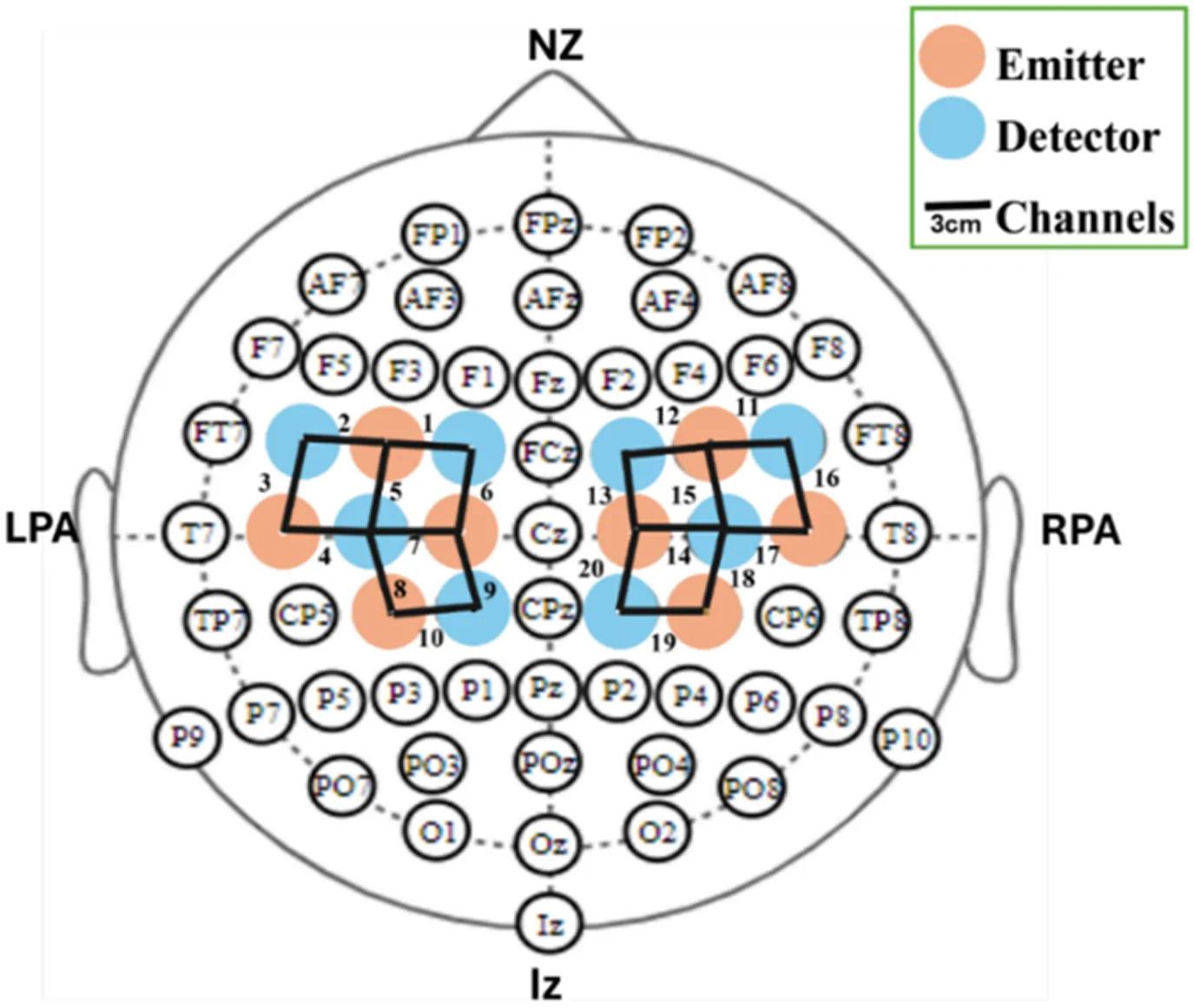
Arrangement of the optodes onto the motor cortex according to the 10 to 20 international system. Blackline represents the channels in between the orange emitter and blue color detectors, which are separated by 3 cm.

Emitters and detectors are positioned onto the motor cortex for this experimental data collection, with a separation of 3 cm from one another. According to the literature, 3 cm is the optimal distance to effectively measure the hemodynamic changes in the cerebral cortex. The arrangement of the optodes (montage) is presented in Fig. 2, which creates a total of twenty channels (emitter-detector pair). The NIRSports2 system is provided with Aurora software to acquire the fNIRS signal in the form of light intensity.

### 2.4 Signal Acquisition and Processing

The NIRSport2 system is equipped with two (760 and 850 nm) wavelengths of near-infrared light, which penetrate brain tissue and correspond to absorption characteristics of oxy- and deoxyhemoglobin. The sampling frequency of the NIRSport2 device is 10.1725 Hz, and the acquired signal is in the form of light intensity.

**Fig. 3:**
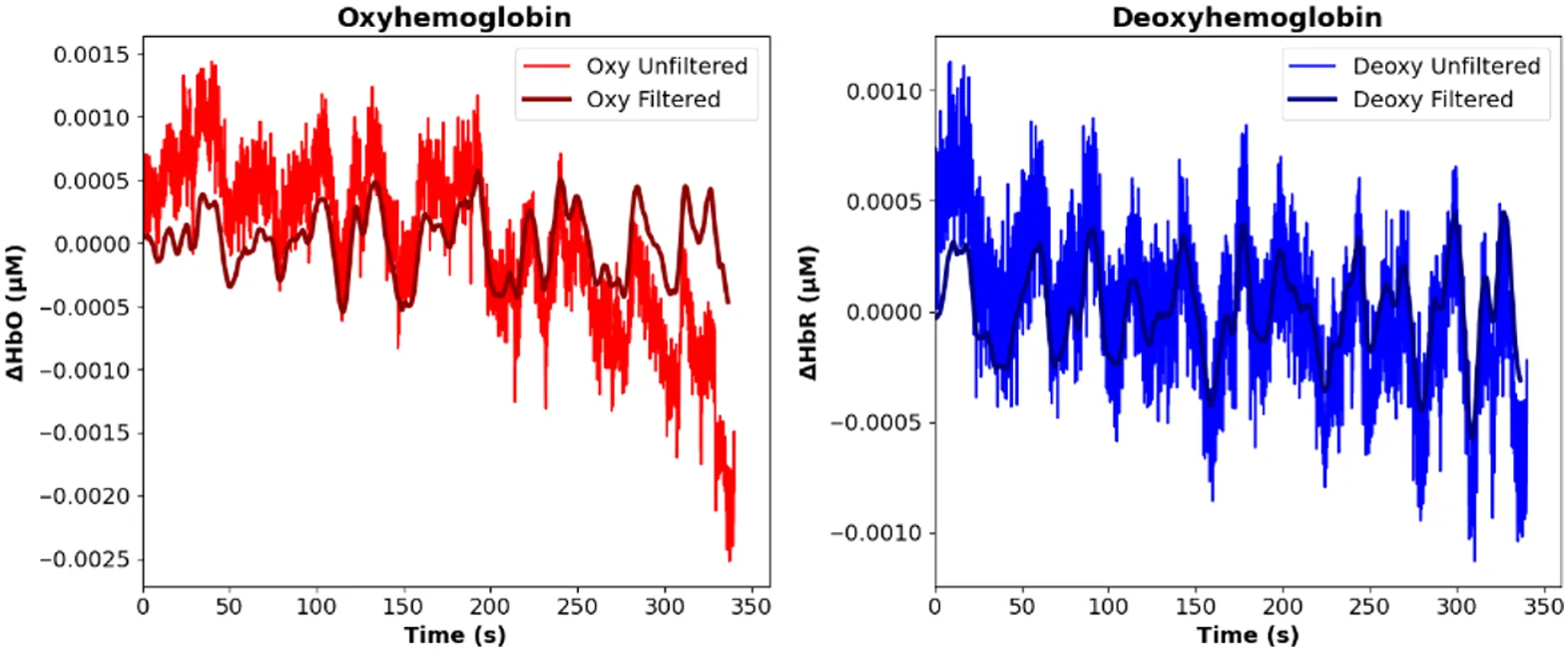
Filtered and unfiltered oxy- and deoxyhemoglobin changes from subject 1, channel 1, with task design overlaid showing task and rest periods and corresponding HbO and HbR hemodynamic responses.

**Fig. 4:**
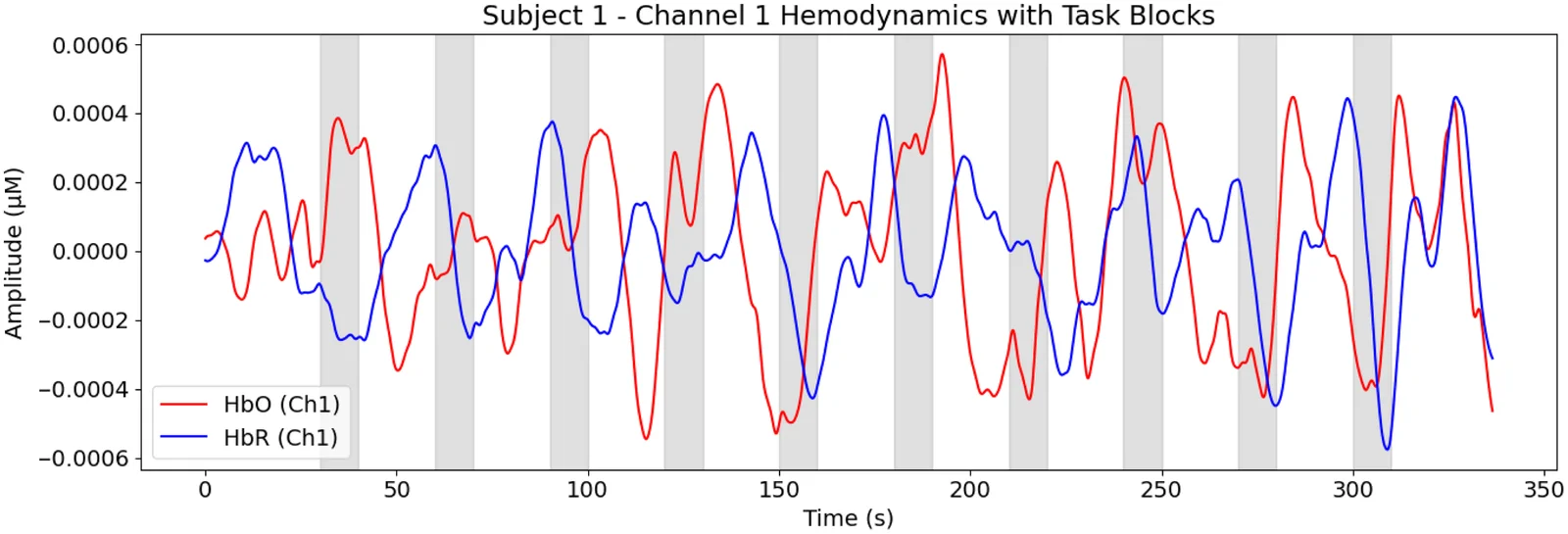
Comparison of HbO and HbR responses for motor task conditions (subject 1, channel 1), showing task-related increases in HbO and decreases in HbR compared with rest.

The nirsLAB (Version: v201904_64bit) software is used to filter out the physiological noises, which is equipped with tools to enhance the reliability and quality of the signal. To eliminate potential noise sources (cardiac and respiratory artifacts) and motion-related interference, bandpass filters with a cutoff frequency of 0.01 and 0.3 Hz are applied. Following this, MBLL converts the optical density dataset into the change in oxy- and deoxyhemoglobin concentration values. Filtered and unfiltered oxy- and deoxyhemoglobin signals are shown in Figs. 3 and 4, and Eq. (1) represents MBLL and is used to convert the optical density ($\Delta A(t;\lambda_{2})$) into change in oxy- and deoxyhemoglobin [ΔHbO(t), ΔHbR(t)].^25^

$$[\begin{array}{c} \Delta \mathrm{HbO}(t)\\ \Delta \mathrm{HbR}(t) \end{array}]=\frac{{\left[\begin{array}{cc} \epsilon_{\mathrm{HbO}}\left(\lambda_{1}\right) & \epsilon_{\mathrm{HbR}}\left(\lambda_{1}\right)\\ \epsilon_{\mathrm{HbO}}\left(\lambda_{2}\right) & {\mathrm{\epsilon ;}}_{\mathrm{HbR}}\left(\lambda_{2}\right) \end{array}\right]}^{-1}[\begin{array}{c} \Delta A(t;\lambda_{1})\\ \Delta A(t;\lambda_{2}) \end{array}]}{l\times d}$$ (1)

ΔHbR(t) and ΔHbO(t) represent the change in concentration of deoxy- and oxyhemoglobin in (μM). $\epsilon_{\mathrm{HbO}}(\lambda ),\;\epsilon_{\mathrm{HbR}}(\lambda )$ extinction coefficients in $\left({\mathrm{\mu M}}^{-1}\;{\mathrm{cm}}^{-1}\right)$. $A\;(t;\lambda_{1}),A\;(t;\lambda_{2})$ the optical density at two different wavelengths $\lambda_{1}$ and $\lambda_{2}$. l represent the separation between the detector and source (3 cm). d represents the mean differential path length factor (DPF). The DPF was set to 7.25 for the 760 nm wavelength and 6.38 for the 850 nm wavelength.^26^

## 3 Dataset Details

**Fig. 5:**
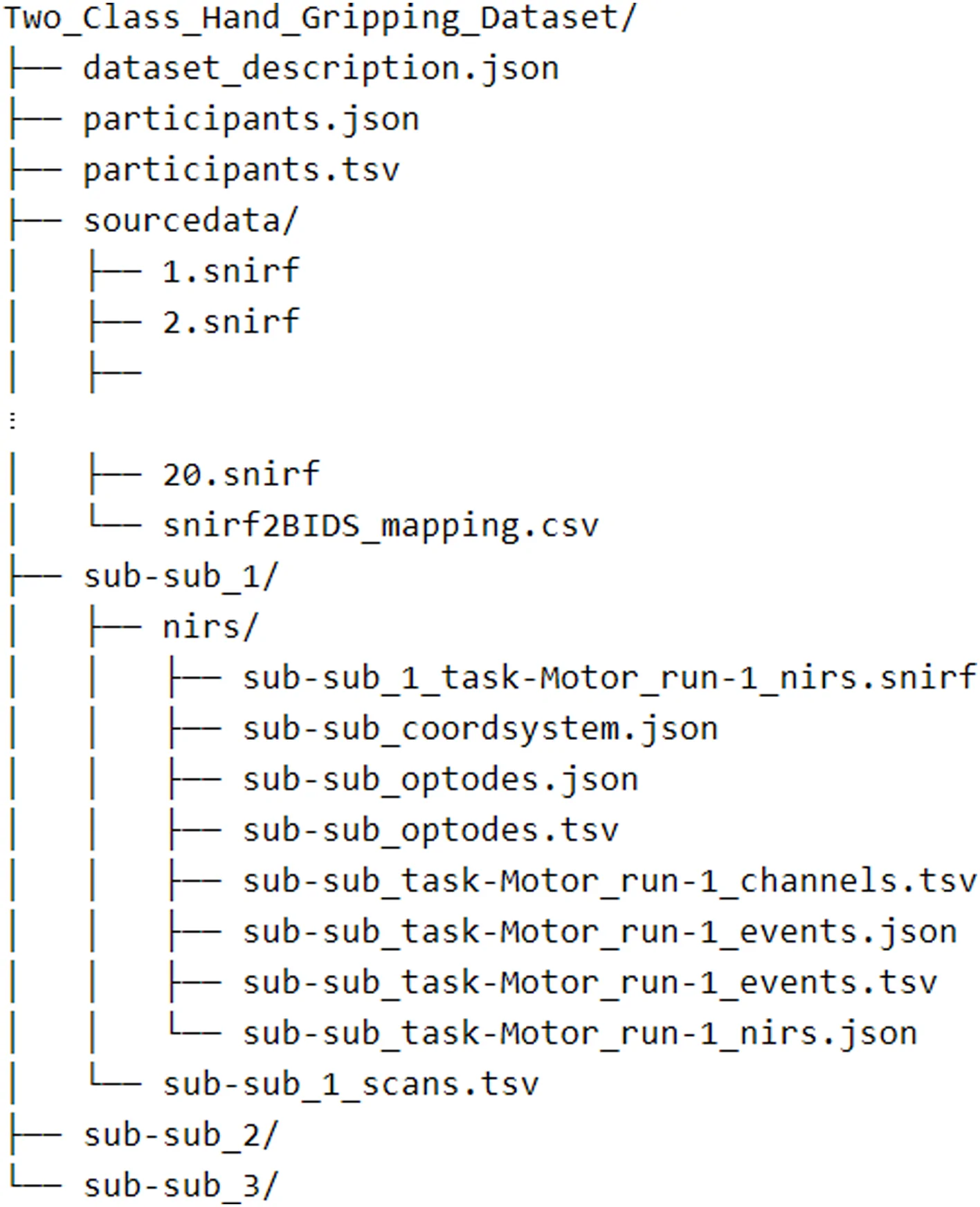
Directory structure of the Figshare dataset showing the organization of raw fNIRS data files. The figure illustrates the hierarchical folder arrangement, including source data, subject-wise directories, and associated metadata, signal, and event files, which are provided to support standardized and reproducible analysis.

Figure 5 represents the folder and subfolder structure of the dataset uploaded to Figshare, including the raw fNIRS data files and associated metadata and BIDS-related files.

## Data Availability

The data is publicly available on the Figshare database, with files organized as illustrated in Fig. 5. The common archive contains raw fNIRS data as well as related metadata files needed to perform standardized and reproducible analysis.^27^ Preprocessed files are provided as .txt files at https://figshare.com/s/f6a48151d9348be9cf5a.
